# Role of Biological Sex in the Cardiovascular-Gut Microbiome Axis

**DOI:** 10.3389/fcvm.2021.759735

**Published:** 2022-01-10

**Authors:** Shuangyue Li, Georgios Kararigas

**Affiliations:** ^1^State Key Laboratory of Cardiovascular Diseases, National Center for Cardiovascular Diseases, Fuwai Hospital, Chinese Academy of Medical Sciences and Peking Union Medical College, Beijing, China; ^2^Department of Physiology, Faculty of Medicine, University of Iceland, Reykjavík, Iceland

**Keywords:** cardiovascular, gut microbiota, heart failure, sex differences, vasculature

## Abstract

There has been a recent, unprecedented interest in the role of gut microbiota in host health and disease. Technological advances have dramatically expanded our knowledge of the gut microbiome. Increasing evidence has indicated a strong link between gut microbiota and the development of cardiovascular diseases (CVD). In the present article, we discuss the contribution of gut microbiota in the development and progression of CVD. We further discuss how the gut microbiome may differ between the sexes and how it may be influenced by sex hormones. We put forward that regulation of microbial composition and function by sex might lead to sex-biased disease susceptibility, thereby offering a mechanistic insight into sex differences in CVD. A better understanding of this could identify novel targets, ultimately contributing to the development of innovative preventive, diagnostic and therapeutic strategies for men and women.

## Introduction

**Figure 1:**
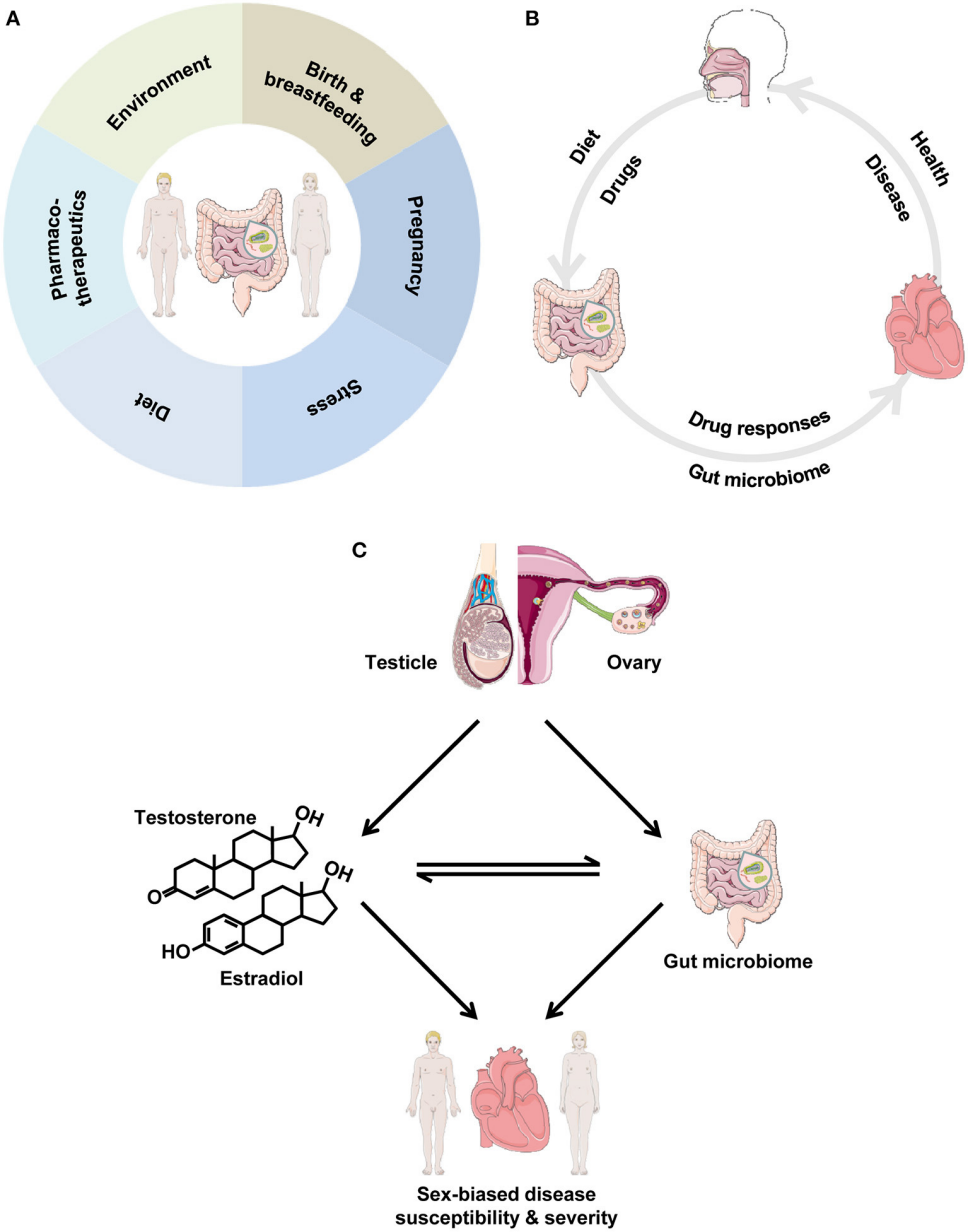
**(A)** Factors impacting the gut microbiome in a sex-specific manner. The depicted factors can lead to marked differences between men and women in the intestinal microbial community. **(B)** The gut microbiome and the cardiovascular system. Dietary and other factors impact the gut microbiome, which, in turn, influences cardiovascular health. In disease, drugs elicit responses in the gut microbiome, which, in turn, influences the progression of cardiovascular disease. **(C)** Interrelationships between biological sex, gut microbiota and the cardiovascular system. We propose the cardiovascular-gut microbiome-sex axis, where the cross-talk between gut microbiota and sex impacts cardiovascular health and disease. Gonadal sex gives rise to male-female hormones and impacts the gut microbiome. In turn, there is a close, bi-directional interaction between sex hormones and gut microbiota, as they influence each other, thereby leading to sex-biased disease susceptibility and severity, ultimately leading to the observed sex differences in cardiovascular disease.

Despite advances in prevention strategies, as well as pharmacological and technology-based cardiovascular (CV) therapies, CV diseases (CVD) remain a major health burden, as they are the leading cause of morbidity and mortality (1). Notably, the development, progression and outcome of CVD, as well as the response to CV pharmacotherapies differ significantly between the sexes (2, 3). However, the contributing mechanisms are incompletely understood. Important modifiable risk factors for CVD include diabetes, hyperlipidemia, hypertension and obesity (4). These factors are linked to nutrition, and interventions aiming at modifying dietary patterns are expected to be beneficial in their successful management and the prevention of CVD (5–8). Interestingly, the old Chinese proverb “disease enters from the mouth” appears to be of relevance. The gut microbiome and its involvement in the development and outcome of CVD have recently attracted wide interest (9, 10), thereby emerging as a key modulator of CV health and disease. Of note, there are several factors that can alter the gut microbiome in a sex-specific manner (Figure 1A). In the present article, we highlight how the gut microbiome can affect the CV system and we explore how biological sex and sex hormones may influence this interaction, thereby contributing to sex differences in CVD. It is not our purpose to provide an exhaustive analysis of the interplay between gut microbiota and CVD, but rather we identify important examples, where biological sex may be of relevance.

## Gut Microbiome

The human gut hosts tens of thousands of microorganisms, up to 100 trillion microbes, which are collectively referred to as the gut microbiome (11, 12). The four dominant bacterial phyla in the human gut are Actinobacteria, Bacteroidetes, Firmicutes and Proteobacteria (13), and the Bacteroidetes and Firmicutes phyla constitute the vast majority of the dominant human gut microbiota (14). There is considerable microbial diversity among different individuals because of variations in age, genetics, geography, hygiene, nutrition and social behaviors (15, 16). The gut microbes have an important role in human health and disease, affecting body weight and digestion, the protection against infection, the risk of autoimmune diseases, as well as the body's response to drugs (17–21). In this context, perturbations of the intestinal microbial community through dietary and environmental factors, as well as genetic factors, among others, can lead to the development of various diseases, including autism, autoimmune diseases, cancer, CVD, diabetes, obesity and other diseases (10, 18, 22–30). Technological advances in the genomic and metabonomic fields, as well as in the assessment of the composition of the intestinal microbiome, have improved our knowledge and understanding of the complex interaction of the gut microbiome with the CV system. Recent findings highlight the potential benefit of developing microbiome-targeted therapies for CVD prevention and treatment, thereby pointing to possible applications in personalized medicine (31, 32).

## Overview of the Interplay Between the gut Microbiome and the CV System

Alterations in gut microbiota influence the CV system and can lead to the development of CVD (Figure 1B). The intestinal microbiota produce a large number of metabolites, some of which are absorbed into the systemic circulation and play a bioactive role, while others are further metabolized by host enzymes and thus become agents of microbial influence on the host CV system (9, 10, 33). As a result, the intestinal tract with its microbes can act as an “endocrine-like” organ, impacting the distal CV target organs through a variety of metabolism-related processes, thereby affecting the onset and progression of CVD. In this section, we highlight important examples with particular emphasis on how this interaction might impinge on the development of CVD.

### Hypertension

Hypertension is a major risk factor for a variety of CVD (34). Gut dysbiosis—an imbalance of gut microbiota—has been linked to hypertension. Significant changes in the gut microbiome have been reported in human hypertensive patients and different hypertensive animal models, including spontaneously hypertensive rats, Dahl salt-sensitive rats, the chronic angiotensin II infusion rat model and high-salt diet-fed mice (35–38). In particular, a dysbiotic pattern was reported in spontaneously hypertensive rats and the chronic angiotensin II infusion rat model. This included decreased microbial richness and an increased Firmicutes:Bacteroidetes ratio, thereby leading to decreases in bacteria that produce the short-chain fatty acids (SCFA) acetate and butyrate (35). SCFA are major bioactive metabolites of gut microbiota, by which the host's biological processes and distal organs can be influenced. In this context, SCFA can regulate blood pressure by acting on various G protein coupled receptors (39). Transplantation studies have indicated a causal relationship between gut dysbiosis and hypertension. Transplant of cecal contents from hypertensive rats into normotensive rats led to increased systolic blood pressure, as well as decreased microbial richness and an increased Firmicutes:Bacteroidetes ratio (40, 41). Furthermore, fecal transplantation from hypertensive human patients to germ-free mice led to increased blood pressure in the mice (38). Mouse studies showed that a diet high in fiber increased the prevalence of *Bacteroides acidifaciens* compared with mice fed a control diet (42). This was associated with increased levels of one of the main metabolites of the gut microbiota, the SCFA acetate, which may confer protection against the development of hypertension (42). In contrast, high salt intake, which can lead to hypertension, depleted Lactobacillus murinus in mice, and treatment of mice with L. murinus prevented salt-sensitive hypertension by modulating T helper 17 cells (36). Along this line, a moderate high-salt challenge in a pilot study in humans reduced intestinal survival of Lactobacillus spp., increased T helper 17 cells and increased blood pressure (36). Collectively, these data reveal the gut microbiota as a crucial regulator of blood pressure and indicate that dietary interventions may be useful in manipulating intestinal microbial composition and function to protect against hypertension.

### Atherosclerosis

Atherosclerosis is a leading cause of various CVD, including coronary artery disease, stroke and peripheral artery disease. Earlier studies suggested that bacteria from the gut may correlate with disease markers of atherosclerosis (43). Since then, a number of studies has shown that the gut microbiome plays an important role in a variety of atherosclerotic conditions (44–48). A mechanistic link between gut microbiota and the development of atherosclerosis has been established. In particular, bacteria in the gut metabolize dietary phosphatidylcholine to trimethylamine, which crosses the gut-epithelial barrier and is then carried to the liver, where it is subsequently metabolized to the proatherogenic molecule trimethylamine-N-oxide (TMAO) (49–51). Importantly, plasma TMAO levels have been associated with mortality in patients with stable coronary artery disease, as well as in patients with peripheral artery disease, independent of traditional risk factors (52, 53). In an experimental model of myocardial infarction, decreased left ventricular function was underlain by increased gut permeability, mediated by tight junction protein suppression, and microbial translocation, thereby triggering systemic inflammation, ultimately contributing to the pathogenesis of CVD (54). Notably, probiotic administration conferred cardioprotective effects, including improved left ventricular function (55, 56). Collectively, these data demonstrate a key role of gut microbiota in the development of atherosclerosis and suggest that the use of probiotics, in addition to standard drug therapies, may provide additional benefits for patients with coronary artery disease, myocardial infarction and other atherosclerotic conditions.

### Heart Failure

Heart failure (HF) is a devastating syndrome with poor prognosis. Gut microbiota and their metabolites produced from dietary metabolism have been linked to HF. To this extent, gut dysbiosis with decreased microbial richness and increased gut permeability has been reported in patients with HF (57, 58), as well as in mice with pressure overload-induced HF (59). Further studies in humans have shown that the gut microbiome-derived metabolite TMAO is increased in HF and that its levels are associated with poor prognosis (60–64). Studies with experimental animals have suggested a causal role of TMAO in the development of HF. In particular, treatment of rodents with TMAO led to myocardial hypertrophy and maladaptive remodeling, including ventricular dilation and wall thinning, systolic dysfunction and increased fibrosis (65, 66). Collectively, these data suggest a key role of gut microbiota and their metabolites in the pathogenesis and progression of HF.

## Brief Overview of Sex Differences in CVD

There are pronounced differences between men and women in the epidemiology, manifestation, pathophysiology, treatment and prognosis of CVD. As these have been reviewed recently elsewhere (3, 76, 77), a brief overview is provided here. The incidence of CVD differs significantly between men and women; for example, women have a greater risk of developing Takotsubo cardiomyopathy than men (78). The association of major modifiable risk factors with incident myocardial infarction differs between the sexes and age influences this interaction significantly (79, 80). Consequently, the younger age of onset of acute myocardial infarction in men (~10 years earlier than women) is largely explained by higher levels of risk factors, including abnormal lipids and smoking (79). In addition, women may have risk factors that are unique to them, such as preeclampsia (high blood pressure during pregnancy) and gestational diabetes, thereby increasing the risk of CVD in female individuals only. Women are also more likely to present with HF with preserved ejection fraction (81, 82), which may be due to sex-biased remodeling of myocardial extracellular matrix (83), and the decline of estrogen at menopause might contribute to its pathogenesis (84). Along this line, under pressure overload, there is a higher proportion of male patients with increased left ventricular mass and end-diastolic diameter, and decreased left ventricular relative wall thickness and function (85–91), associated with greater activation of inflammatory factors (92, 93). Physiological differences between men and women may also lead to sex differences in the response to treatment (2, 94–97). Overall, there are significant sex differences in the outcome of a variety of CV disorders (98–100).

## Sex Differences in the Composition of the Gut Microbiome

Human and animal studies have shown that biological sex has an impact on gut microbial composition (Table 1). In particular, significant differences in the composition of gut microbiota between healthy men and women have been reported, with women appearing to have lower Bacteroidetes abundance compared with men (67–70). The gut microbe-sex interaction, however, is, not surprisingly, affected by obesity, as the abundance of the Bacteroides genus was reported to be lower in men than in women with a body mass index >33 (71). A study of different strains of mice showed that when considering the data at the single strain level, several taxa exhibited significant differences in abundance between the sexes (101). Among these, Bacteroidetes abundance is lower in female mice compared with male mice (72). Interestingly, sex differences in the gut microbiome appear to be responsible for hormone-dependent regulation of autoimmune disease (19). Along this line, in an experimental model of colitis, sex differences in gut microbiota composition were associated with sex-biased severity of the disease (73). Studies in rodents have shown that the levels of the gut microbiome-derived metabolite TMAO are higher in females than in males (74, 102). Biological sex also influences the interaction between the gut microbiome and environmental factors, such as diet (75, 101, 103, 104). For example, the interaction between dietary habits, such as yogurt consumption, and the gut microbiota is significantly different between healthy young male and female adults (105). Collectively, these data demonstrate that microbial composition and diversity differ significantly between the sexes. In this context, we put forward that differences in microbial composition and function between men and women may be a contributing factor to the observed sex differences in CVD (Figure 1C).

**Table 1:** Sex differences in the gut microbiome.

**Component**	**Sex difference**	**References**
Bacteroides	healthy men > healthy women	(67–70)
Prevotella	healthy men > healthy women	(69)
Bacteroides	obese men < obese women	(71)
Firmicutes	obese men < obese women	(71)
Bacteroides	healthy male mice > healthy female mice	(72)
Ruminococcaceae	healthy male mice > healthy female mice	(73)
Peptostreptococcaceae	healthy male mice < healthy female mice	(73)
TMAO	healthy male mice < healthy female mice	(74)
SCFA	male > female rats fed an oligofructose-supplemented diet	(75)

*SCFA, short-chain fatty acids; TMAO, trimethylamine-N-oxide*.

## Regulation of the Gut Microbiome by Sex Hormones

A large body of literature demonstrates that sex hormones modulate CV physiology and pathology. Of relevance, among the various factors underlying sex differences in CVD, such as sex chromosomes and (epi)genetic factors (3, 106), sex hormones appear to be crucial. For example, the steroid hormone 17β-estradiol (E2) and its receptors (ER) are thought to play a major role (107–111). The E2/ER axis has been shown to have vast effects in the CV system, regulating, for example, contractile function (micro), vascular function, metabolic processes, calcium signaling, gene expression and protein abundance (112–126), which can be sex-dependent (109, 127–132).

Along this line, among various types of hormones with regulatory effects, sex hormones have a key role in the regulation of the distribution of the gut microbiome. In this context, sex hormones affect gene expression and other processes of gut microbiota, thereby influencing intestinal microbial composition and function. In parallel, the microbial community also alters sex hormone levels, thereby regulating disease development and outcome (19, 72). Therefore, there is a complex interaction between gut microbiota and sex hormones. Interestingly, this is true not only for mammals but also for fish. Treatment of zebrafish with E2 altered the intestinal microbial composition significantly, which may lead to further physiological changes in the host (133). Androgens also appear to influence the composition of the gut microbiome. In fact, in pathological situations of hormonal excess, such as polycystic ovary syndrome, there is decreased microbial diversity and changes in microbial composition that are associated with metabolic dysregulation, thereby indicating that hyperandrogenism may be linked with gut dysbiosis in women with this disorder (134–137). Moreover, testosterone treatment after gonadectomy prevented the significant changes in gut microbiota composition that occurred in untreated males (101). Further studies in humans have shown that the composition of gut microbiota differs significantly among women with different hormonal status (67). The key role of sex hormones in microbial composition is further supported by studies in mice with gonadectomy, which resulted in an altered gut microbiome (101). To this extent, it was shown that the deficiency of androgens alters the intestinal microbiome and induces abdominal obesity in a diet-dependent manner (138). Collectively, these data indicate that sex hormones mediate (at least partly) the differences in gut microbiota composition between the sexes. Currently, it is unclear how sex hormones regulate the composition and function of the gut microbial community. Further research is warranted.

## Challenges and Opportunities

Potential differences in microbial composition, diversity and function between humans and animal models might limit the translation of experimental studies with animals. Furthermore, given the major impact that environmental factors have on gut microbiota, it needs to be highlighted that most environmental conditions of humans differ significantly from those of laboratory animals. In addition, estrous cycle-related changes in gut microbiota in rodents might not reflect any changes due to menstruation in women. Nevertheless, the degree of any inconsistencies remains to be determined. Lastly, abundant species do not necessarily confer abundant molecular functions, underscoring the importance of a functional analysis to understand microbial communities (139).

The role of sex has yet underestimated consequences for physiology and pathology (140). A better understanding of the mechanisms accounting for sex differences in microbial composition could provide opportunities for the development of novel sex-specific diagnostic tests and therapeutic approaches for CVD. Given the evidence provided by the current literature, we put forward that the “gut microbiome status” in the context of the patient's sex should be taken into account in CVD management and we provide the following specific recommendations as a paradigm: (1) a dietary intervention to modulate gut microbiota could be an innovative nutritional therapeutic strategy for hypertension; (2) a new therapeutic intervention by probiotics targeting gut bacteria and protecting gut function may be a potential option to improve CV outcomes post-myocardial infarction; (3) strategies for the modulation of TMAO levels could be beneficial in the prevention of HF; (4) approaches to reduce the levels of TMAO in patients with HF could improve long-term prognosis. In this context, an experimental approach for the directed remodeling of the mouse gut microbiome with peptide treatment was recently shown to inhibit the development of atherosclerosis (141).

Moreover, the gut has been shown to be a target of severe acute respiratory syndrome coronavirus 2 (SARS-CoV-2). In fact, gastrointestinal manifestations of SARS-CoV-2, such as anorexia, nausea, vomiting, diarrhea and hepato-cellular injury (transaminitis), among others, have been widely reported (142, 143). SARS-CoV-2 is primarily considered a respiratory pathogen. Nevertheless, patients with coronavirus disease 2019 (COVID-19) present with gastrointestinal symptoms, given that angiotensin-converting enzyme 2 (ACE2), which SARS-CoV-2 uses as a host cell entry receptor (144), is ubiquitously present in the human gut, including small intestine and colonic enterocytes, hepatocytes and cholangiocytes. However, the effects and impact of SARS-CoV-2 on host microbial flora and gut microbiota composition are unclear. Interestingly, COVID-19 patients appear to have sex-dependent CV risk and complications. However, the underlying mechanisms are incompletely understood (145, 146). To this extent, we put forward the notion that SARS-CoV-2 may lead to significant sex differences in the gut microbiome status of COVID-19 patients, which, in turn, impacts the CV system, thereby contributing to the observed sex-biased CV complications in these patients. This further highlights the opportunity of employing the gut microbiome as a novel target for sex-specific therapeutic interventions for (severe) CV complications in patients with COVID-19. Further research is warranted.

## Conclusions

The mechanisms by which the gut microbiome affects CV (patho) physiology are incompletely understood. From several human and animal studies, it is clear that the gut microbiome exerts sex-biased effects in health and disease. At least in part, sex hormones account for these sex-dependent effects in a complex bi-directional interaction with the microbial community. Gut microbiota may become a novel target for pharmacological or dietary interventions as part of new preventive and therapeutic strategies in CVD. A better understanding of the effects of biological sex and considering its role in such novel approaches will improve clinical care and management via new strategies for the prevention, diagnosis and treatment of disease in both men and women.

## Author Contributions

GK conceived the work. SL and GK wrote the manuscript. Both authors contributed to the article and approved the submitted version.

## Funding

GK acknowledges lab support provided by grants from the Icelandic Research Fund (217946-051), Icelandic Cancer Society Research Fund and University of Iceland Research Fund.

## Conflict of Interest

The authors declare that the research was conducted in the absence of any commercial or financial relationships that could be construed as a potential conflict of interest.

## Publisher's Note

All claims expressed in this article are solely those of the authors and do not necessarily represent those of their affiliated organizations, or those of the publisher, the editors and the reviewers. Any product that may be evaluated in this article, or claim that may be made by its manufacturer, is not guaranteed or endorsed by the publisher.

## References

[1] Collaborators. GCoD. Global, regional, and national age-sex-specific mortality for 282 causes of death in 195 countries and territories, 1980-2017: a systematic analysis for the global burden of disease study 2017. Lancet. (2018) 392:1736–88. 10.1016/S0140-6736(18)32203-7 30496103 PMC6227606

[2] Gaignebet L Kararigas G. En route to precision medicine through the integration of biological sex into pharmacogenomics. Clin Sci. (2017) 131:329–42. 10.1042/CS20160379 28159880

[3] Regitz-Zagrosek V Kararigas G. Mechanistic pathways of sex differences in cardiovascular disease. Physiol Rev. (2017) 97:1–37. 10.1152/physrev.00021.2015 27807199

[4] Yusuf S Joseph P Rangarajan S Islam S Mente A Hystad P. Modifiable risk factors, cardiovascular disease, and mortality in 155 722 individuals from 21 high-income, middle-income, and low-income countries (PURE): a prospective cohort study. Lancet. (2020) 395:795–808. 10.1016/S0140-6736(19)32008-2 31492503 PMC8006904

[5] Mirzaei H Di Biase S Longo VD. Dietary interventions, cardiovascular aging, and disease: animal models and human studies. Circ Res. (2016) 118:1612–25. 10.1161/CIRCRESAHA.116.307473 27174953

[6] Ley SH Hamdy O Mohan V Hu FB. Prevention and management of type 2 diabetes: dietary components and nutritional strategies. Lancet. (2014) 383:1999–2007. 10.1016/S0140-6736(14)60613-9 24910231 PMC4751088

[7] Mozaffarian D. Dietary and policy priorities for cardiovascular disease, diabetes, and obesity: a comprehensive review. Circulation. (2016) 133:187–225. 10.1161/CIRCULATIONAHA.115.018585 26746178 PMC4814348

[8] Bechthold A Boeing H Schwedhelm C Hoffmann G Knuppel S Iqbal K. Food groups and risk of coronary heart disease, stroke and heart failure: a systematic review and dose-response meta-analysis of prospective studies. Crit Rev Food Sci Nutr. (2019) 59:1071–90. 10.1080/10408398.2017.1392288 29039970

[9] Tang WH Kitai T Hazen SL. Gut microbiota in cardiovascular health and disease. Circ Res. (2017) 120:1183–96. 10.1161/CIRCRESAHA.117.309715 28360349 PMC5390330

[10] Tang WH Hazen SL. The gut microbiome and its role in cardiovascular diseases. Circulation. (2017) 135:1008–10. 10.1161/CIRCULATIONAHA.116.024251 28289004 PMC5354081

[11] Ley RE Peterson DA Gordon JI. Ecological and evolutionary forces shaping microbial diversity in the human intestine. Cell. (2006) 124:837–48. 10.1016/j.cell.2006.02.017 16497592

[12] Eckburg PB Bik EM Bernstein CN Purdom E Dethlefsen L Sargent M. Diversity of the human intestinal microbial flora. Science. (2005) 308:1635–8. 10.1126/science.1110591 15831718 PMC1395357

[13] Khanna S Tosh PK. A clinician's primer on the role of the microbiome in human health and disease. Mayo Clin Proc. (2014) 89:107–14. 10.1016/j.mayocp.2013.10.011 24388028

[14] Zoetendal EG Rajilic-Stojanovic M de Vos WM. High-throughput diversity and functionality analysis of the gastrointestinal tract microbiota. Gut. (2008) 57:1605–15. 10.1136/gut.2007.133603 18941009

[15] Yatsunenko T Rey FE Manary MJ Trehan I Dominguez-Bello MG Contreras M. Human gut microbiome viewed across age and geography. Nature. (2012) 486:222–7. 10.1038/nature11053 22699611 PMC3376388

[16] Flint HJ. The impact of nutrition on the human microbiome. Nutr Rev. (2012) 70(Suppl. 1):S10–3. 10.1111/j.1753-4887.2012.00499.x 22861801

[17] van Nood E Vrieze A Nieuwdorp M Fuentes S Zoetendal EG de Vos WM. Duodenal infusion of donor feces for recurrent clostridium difficile. N Engl J Med. (2013) 368:407–15. 10.1056/NEJMoa1205037 23323867

[18] Vatanen T Kostic AD d'Hennezel E Siljander H Franzosa EA Yassour M. Variation in microbiome LPS immunogenicity contributes to autoimmunity in humans. Cell. (2016) 165:842–53. 10.1016/j.cell.2016.04.007 27133167 PMC4950857

[19] Markle JG Frank DN Mortin-Toth S Robertson CE Feazel LM Rolle-Kampczyk U. Sex differences in the gut microbiome drive hormone-dependent regulation of autoimmunity. Science. (2013) 339:1084–8. 10.1126/science.1233521 23328391

[20] Gopalakrishnan V Spencer CN Nezi L Reuben A Andrews MC Karpinets TV. Gut microbiome modulates response to anti-PD-1 immunotherapy in melanoma patients. Science. (2018) 359:97–103. 10.1126/science.aan4236 29097493 PMC5827966

[21] Uribe-Herranz M Rafail S Beghi S Gil-de-Gomez L Verginadis I Bittinger K. Gut microbiota modulate dendritic cell antigen presentation and radiotherapy-induced antitumor immune response. J Clin Invest. (2020) 130:466–79. 10.1172/JCI124332 31815742 PMC6934221

[22] Gopalakrishnan V Helmink BA Spencer CN Reuben A Wargo JA. The influence of the gut microbiome on cancer, immunity, and cancer immunotherapy. Cancer Cell. (2018) 33:570–80. 10.1016/j.ccell.2018.03.015 29634945 PMC6529202

[23] Turnbaugh PJ Ley RE Mahowald MA Magrini V Mardis ER Gordon JI. An obesity-associated gut microbiome with increased capacity for energy harvest. Nature. (2006) 444:1027–31. 10.1038/nature05414 17183312

[24] Forslund K Hildebrand F Nielsen T Falony G Le Chatelier E Sunagawa S. Disentangling type 2 diabetes and metformin treatment signatures in the human gut microbiota. Nature. (2015) 528:262–6. 10.1038/nature15766 26633628 PMC4681099

[25] Vuong HE Hsiao EY. Emerging roles for the gut microbiome in autism spectrum disorder. Biol Psychiatry. (2017) 81:411–23. 10.1016/j.biopsych.2016.08.024 27773355 PMC5285286

[26] Hall AB Tolonen AC Xavier RJ. Human genetic variation and the gut microbiome in disease. Nat Rev Genet. (2017) 18:690–9. 10.1038/nrg.2017.63 28824167

[27] McKenzie C Tan J Macia L Mackay CR. The nutrition-gut microbiome-physiology axis and allergic diseases. Immunol Rev. (2017) 278:277–95. 10.1111/imr.12556 28658542

[28] Ghaisas S Maher J Kanthasamy A. Gut microbiome in health and disease: linking the microbiome-gut-brain axis and environmental factors in the pathogenesis of systemic and neurodegenerative diseases. Pharmacol Ther. (2016) 158:52–62. 10.1016/j.pharmthera.2015.11.012 26627987 PMC4747781

[29] Rogers GB Keating DJ Young RL Wong ML Licinio J Wesselingh S. From gut dysbiosis to altered brain function and mental illness: mechanisms and pathways. Mol Psychiatry. (2016) 21:738–48. 10.1038/mp.2016.50 27090305 PMC4879184

[30] Abdollahi-Roodsaz S Abramson SB Scher JU. The metabolic role of the gut microbiota in health and rheumatic disease: mechanisms and interventions. Nat Rev Rheumatol. (2016) 12:446–55. 10.1038/nrrheum.2016.68 27256713

[31] Kurilshikov A van den Munckhof ICL Chen L Bonder MJ Schraa K Rutten JHW. Gut microbial associations to plasma metabolites linked to cardiovascular phenotypes and risk. Circ Res. (2019) 124:1808–20. 10.1161/CIRCRESAHA.118.314642 30971183

[32] Zhernakova DV Le TH Kurilshikov A Atanasovska B Bonder MJ Sanna S. Individual variations in cardiovascular-disease-related protein levels are driven by genetics and gut microbiome. Nat Genet. (2018) 50:1524–32. 10.1038/s41588-018-0224-7 30250126 PMC6241851

[33] Jonsson AL Backhed F. Role of gut microbiota in atherosclerosis. Nat Rev Cardiol. (2017) 14:79–87. 10.1038/nrcardio.2016.183 27905479

[34] Sabbatini AR Kararigas G. Estrogen-related mechanisms in sex differences of hypertension and target organ damage. Biol Sex Differ. (2020) 11:31. 10.1186/s13293-020-00306-7 32487164 PMC7268741

[35] Yang T Santisteban MM Rodriguez V Li E Ahmari N Carvajal JM. Gut dysbiosis is linked to hypertension. Hypertension. (2015) 65:1331–40. 10.1161/HYPERTENSIONAHA.115.05315 25870193 PMC4433416

[36] Wilck N Matus MG Kearney SM Olesen SW Forslund K Bartolomaeus H. Salt-responsive gut commensal modulates TH17 axis and disease. Nature. (2017) 551:585–9. 10.1038/nature24628 29143823 PMC6070150

[37] Mell B Jala VR Mathew AV Byun J Waghulde H Zhang Y. Evidence for a link between gut microbiota and hypertension in the Dahl rat. Physiol Genomics. (2015) 47:187–97. 10.1152/physiolgenomics.00136.2014 25829393 PMC4451389

[38] Li J Zhao F Wang Y Chen J Tao J Tian G. Gut microbiota dysbiosis contributes to the development of hypertension. Microbiome. (2017) 5:14. 10.1186/s40168-016-0222-x 28143587 PMC5286796

[39] Pluznick JL Protzko RJ Gevorgyan H Peterlin Z Sipos A Han J. Olfactory receptor responding to gut microbiota-derived signals plays a role in renin secretion and blood pressure regulation. Proc Natl Acad Sci USA. (2013) 110:4410–5. 10.1073/pnas.1215927110 23401498 PMC3600440

[40] Durgan DJ Ganesh BP Cope JL Ajami NJ Phillips SC Petrosino JF. Role of the gut microbiome in obstructive sleep apnea-induced hypertension. Hypertension. (2016) 67:469–74. 10.1161/HYPERTENSIONAHA.115.06672 26711739 PMC4713369

[41] Adnan S Nelson JW Ajami NJ Venna VR Petrosino JF Bryan RM. Alterations in the gut microbiota can elicit hypertension in rats. Physiol Genomics. (2017) 49:96–104. 10.1152/physiolgenomics.00081.2016 28011881 PMC5336599

[42] Marques FZ Nelson E Chu PY Horlock D Fiedler A Ziemann M. High-fiber diet and acetate supplementation change the gut microbiota and prevent the development of hypertension and heart failure in hypertensive mice. Circulation. (2017) 135:964–77. 10.1161/CIRCULATIONAHA.116.024545 27927713

[43] Koren O Spor A Felin J Fak F Stombaugh J Tremaroli V. Human oral, gut, and plaque microbiota in patients with atherosclerosis. Proc Natl Acad Sci USA. (2011) 108(Suppl. 1):4592–8. 10.1073/pnas.1011383107 20937873 PMC3063583

[44] Jie Z Xia H Zhong SL Feng Q Li S Liang S. The gut microbiome in atherosclerotic cardiovascular disease. Nat Commun. (2017) 8:845. 10.1038/s41467-017-00900-1 29018189 PMC5635030

[45] Emoto T Yamashita T Sasaki N Hirota Y Hayashi T So A. Analysis of gut microbiota in coronary artery disease patients: a possible link between gut microbiota and coronary artery disease. J Atheroscler Thromb. (2016) 23:908–21. 10.5551/jat.32672 26947598 PMC7399299

[46] Emoto T Yamashita T Kobayashi T Sasaki N Hirota Y Hayashi T. Characterization of gut microbiota profiles in coronary artery disease patients using data mining analysis of terminal restriction fragment length polymorphism: gut microbiota could be a diagnostic marker of coronary artery disease. Heart Vessels. (2017) 32:39–46. 10.1007/s00380-016-0841-y 27125213

[47] Yoshida N Emoto T Yamashita T Watanabe H Hayashi T Tabata T. Bacteroides vulgatus and bacteroides dorei reduce gut microbial lipopolysaccharide production and inhibit atherosclerosis. Circulation. (2018) 138:2486–98. 10.1161/CIRCULATIONAHA.118.033714 30571343

[48] Liu H Chen X Hu X Niu H Tian R Wang H. Alterations in the gut microbiome and metabolism with coronary artery disease severity. Microbiome. (2019) 7:68. 10.1186/s40168-019-0683-9 31027508 PMC6486680

[49] Koeth RA Levison BS Culley MK Buffa JA Wang Z Gregory JC. gamma-Butyrobetaine is a proatherogenic intermediate in gut microbial metabolism of L-carnitine to TMAO. Cell Metab. (2014) 20:799–812. 10.1016/j.cmet.2014.10.006 25440057 PMC4255476

[50] Wang Z Klipfell E Bennett BJ Koeth R Levison BS Dugar B. Gut flora metabolism of phosphatidylcholine promotes cardiovascular disease. Nature. (2011) 472:57–63. 10.1038/nature09922 21475195 PMC3086762

[51] Tang WH Wang Z Levison BS Koeth RA Britt EB Fu X. Intestinal microbial metabolism of phosphatidylcholine and cardiovascular risk. N Engl J Med. (2013) 368:1575–84. 10.1056/NEJMoa1109400 23614584 PMC3701945

[52] Senthong V Wang Z Li XS Fan Y Wu Y Tang WH. Intestinal microbiota-generated metabolite trimethylamine-N-Oxide and 5-year mortality risk in stable coronary artery disease: the contributory role of intestinal microbiota in a COURAGE-like patient cohort. J Am Heart Assoc. (2016) 5:e002816. 10.1161/JAHA.115.002816 27287696 PMC4937244

[53] Senthong V Wang Z Fan Y Wu Y Hazen SL Tang WH. Trimethylamine n-oxide and mortality risk in patients with peripheral artery disease. J Am Heart Assoc. (2016) 5:e004237. 10.1161/JAHA.116.004237 27792653 PMC5121520

[54] Zhou X Li J Guo J Geng B Ji W Zhao Q. Gut-dependent microbial translocation induces inflammation and cardiovascular events after ST-elevation myocardial infarction. Microbiome. (2018) 6:66. 10.1186/s40168-018-0441-4 29615110 PMC5883284

[55] Gan XT Ettinger G Huang CX Burton JP Haist JV Rajapurohitam V. Probiotic administration attenuates myocardial hypertrophy and heart failure after myocardial infarction in the rat. Circ Heart Fail. (2014) 7:491–9. 10.1161/CIRCHEARTFAILURE.113.000978 24625365

[56] Tang TWH Chen HC Chen CY Yen CYT Lin CJ Prajnamitra RP. Loss of gut microbiota alters immune system composition and cripples postinfarction cardiac repair. Circulation. (2019) 139:647–59. 10.1161/CIRCULATIONAHA.118.035235 30586712

[57] Luedde M Winkler T Heinsen FA Ruhlemann MC Spehlmann ME Bajrovic A. Heart failure is associated with depletion of core intestinal microbiota. ESC Heart Fail. (2017) 4:282–90. 10.1002/ehf2.12155 28772054 PMC5542738

[58] Pasini E Aquilani R Testa C Baiardi P Angioletti S Boschi F. Pathogenic gut flora in patients with chronic heart failure. JACC Heart Fail. (2016) 4:220–7. 10.1016/j.jchf.2015.10.009 26682791

[59] Boccella N Paolillo R Coretti L D'Apice S Lama A Giugliano G. Transverse aortic constriction induces gut barrier alterations, microbiota remodeling and systemic inflammation. Sci Rep. (2021) 11:7404. 10.1038/s41598-021-86651-y 33795775 PMC8016915

[60] Hayashi T Yamashita T Watanabe H Kami K Yoshida N Tabata T. Gut microbiome and plasma microbiome-related metabolites in patients with decompensated and compensated heart failure. Circ J. (2018) 83:182–92. 10.1253/circj.CJ-18-0468 30487369

[61] Cui X Ye L Li J Jin L Wang W Li S. Metagenomic and metabolomic analyses unveil dysbiosis of gut microbiota in chronic heart failure patients. Sci Rep. (2018) 8:635. 10.1038/s41598-017-18756-2 29330424 PMC5766622

[62] Tang WH Wang Z Fan Y Levison B Hazen JE Donahue LM. Prognostic value of elevated levels of intestinal microbe-generated metabolite trimethylamine-N-oxide in patients with heart failure: refining the gut hypothesis. J Am Coll Cardiol. (2014) 64:1908–14. 10.1016/j.jacc.2014.02.617 25444145 PMC4254529

[63] Suzuki T Heaney LM Bhandari SS Jones DJ Ng LL. Trimethylamine N-oxide and prognosis in acute heart failure. Heart. (2016) 102:841–8. 10.1136/heartjnl-2015-308826 26869641

[64] Schiattarella GG Sannino A Toscano E Giugliano G Gargiulo G Franzone A. Gut microbe-generated metabolite trimethylamine-N-oxide as cardiovascular risk biomarker: a systematic review and dose-response meta-analysis. Eur Heart J. (2017) 38:2948–56. 10.1093/eurheartj/ehx342 29020409

[65] Li Z Wu Z Yan J Liu H Liu Q Deng Y. Gut microbe-derived metabolite trimethylamine N-oxide induces cardiac hypertrophy and fibrosis. Lab Invest. (2019) 99:346–57. 10.1038/s41374-018-0091-y 30068915

[66] Organ CL Otsuka H Bhushan S Wang Z Bradley J Trivedi R. Choline diet and its gut microbe-derived metabolite, trimethylamine n-oxide, exacerbate pressure overload-induced heart failure. Circ Heart Fail. (2016) 9:e002314. 10.1161/CIRCHEARTFAILURE.115.002314 26699388 PMC4943035

[67] Santos-Marcos JA Rangel-Zuniga OA Jimenez-Lucena R Quintana-Navarro GM Garcia-Carpintero S Malagon MM. Influence of gender and menopausal status on gut microbiota. Maturitas. (2018) 116:43–53. 10.1016/j.maturitas.2018.07.008 30244778

[68] Dominianni C Sinha R Goedert JJ Pei Z Yang L Hayes RB. Sex, body mass index, and dietary fiber intake influence the human gut microbiome. PLoS ONE. (2015) 10:e0124599. 10.1371/journal.pone.0124599 25874569 PMC4398427

[69] Mueller S Saunier K Hanisch C Norin E Alm L Midtvedt T. Differences in fecal microbiota in different European study populations in relation to age, gender, and country: a cross-sectional study. Appl Environ Microbiol. (2006) 72:1027–33. 10.1128/AEM.72.2.1027-1033.2006 16461645 PMC1392899

[70] Ding T Schloss PD. Dynamics and associations of microbial community types across the human body. Nature. (2014) 509:357–60. 10.1038/nature13178 24739969 PMC4139711

[71] Haro C Rangel-Zuniga OA Alcala-Diaz JF Gomez-Delgado F Perez-Martinez P Delgado-Lista J. Intestinal microbiota is influenced by gender and body mass index. PLoS ONE. (2016) 11:e0154090. 10.1371/journal.pone.0154090 27228093 PMC4881937

[72] Yurkovetskiy L Burrows M Khan AA Graham L Volchkov P Becker L. Gender bias in autoimmunity is influenced by microbiota. Immunity. (2013) 39:400–12. 10.1016/j.immuni.2013.08.013 23973225 PMC3822899

[73] Kozik AJ Nakatsu CH Chun H Jones-Hall YL. Age, sex, and TNF associated differences in the gut microbiota of mice and their impact on acute TNBS colitis. Exp Mol Pathol. (2017) 103:311–9. 10.1016/j.yexmp.2017.11.014 29175304

[74] Gavaghan McKee CL Wilson ID Nicholson JK. Metabolic phenotyping of nude and normal (Alpk:ApfCD, C57BL10J) mice. J Proteome Res. (2006) 5:378–84. 10.1021/pr050255h 16457604

[75] Shastri P McCarville J Kalmokoff M Brooks SP Green-Johnson JM. Sex differences in gut fermentation and immune parameters in rats fed an oligofructose-supplemented diet. Biol Sex Differ. (2015) 6:13. 10.1186/s13293-015-0031-0 26251695 PMC4527341

[76] Ventura-Clapier R Dworatzek E Seeland U Kararigas G Arnal JF Brunelleschi S. Sex in basic research: concepts in the cardiovascular field. Cardiovasc Res. (2017) 113:711–24. 10.1093/cvr/cvx066 28472454

[77] Colafella KMM Denton KM. Sex-specific differences in hypertension and associated cardiovascular disease. Nat Rev Nephrol. (2018) 14:185–201. 10.1038/nrneph.2017.189 29380817

[78] Templin C Ghadri JR Diekmann J Napp LC Bataiosu DR Jaguszewski M. Clinical features and outcomes of takotsubo (Stress) cardiomyopathy. N Engl J Med. (2015) 373:929–38. 10.1056/NEJMoa1406761 26332547

[79] Anand SS Islam S Rosengren A Franzosi MG Steyn K Yusufali AH. Risk factors for myocardial infarction in women and men: insights from the INTERHEART study. Eur Heart J. (2008) 29:932–40. 10.1093/eurheartj/ehn018 18334475

[80] Culic V Eterovic D Miric D. Meta-analysis of possible external triggers of acute myocardial infarction. Int J Cardiol. (2005) 99:1–8. 10.1016/j.ijcard.2004.01.008 15721492

[81] Lam CS Carson PE Anand IS Rector TS Kuskowski M Komajda M. Sex differences in clinical characteristics and outcomes in elderly patients with heart failure and preserved ejection fraction: the irbesartan in heart failure with preserved ejection fraction (I-PRESERVE) trial. Circ Heart Fail. (2012) 5:571–8. 10.1161/CIRCHEARTFAILURE.112.970061 22887722 PMC4768740

[82] Beale AL Meyer P Marwick TH Lam CSP Kaye DM. Sex differences in cardiovascular pathophysiology: why women are overrepresented in heart failure with preserved ejection fraction. Circulation. (2018) 138:198–205. 10.1161/CIRCULATIONAHA.118.034271 29986961

[83] Dworatzek E Baczko I Kararigas G. Effects of aging on cardiac extracellular matrix in men and women. Proteomics Clin Appl. (2016) 10:84–91. 10.1002/prca.201500031 26280680

[84] Sabbatini AR Kararigas G. Menopause-related estrogen decrease and the pathogenesis of HFpEF: JACC review topic of the week. J Am Coll Cardiol. (2020) 75:1074–82. 10.1016/j.jacc.2019.12.049 32138968

[85] Aurigemma GP Silver KH McLaughlin M Mauser J Gaasch WH. Impact of chamber geometry and gender on left ventricular systolic function in patients > 60 years of age with aortic stenosis. Am J Cardiol. (1994) 74:794–8. 10.1016/0002-9149(94)90437-5 7942552

[86] Carroll JD Carroll EP Feldman T Ward DM Lang RM McGaughey D. Sex-associated differences in left ventricular function in aortic stenosis of the elderly. Circulation. (1992) 86:1099–107. 10.1161/01.CIR.86.4.1099 1394918

[87] Douglas PS Otto CM Mickel MC Labovitz A Reid CL Davis KB. Gender differences in left ventricle geometry and function in patients undergoing balloon dilatation of the aortic valve for isolated aortic stenosis. NHLBI Balloon Valvuloplasty Registry. Br Heart J. (1995) 73:548–54. 10.1136/hrt.73.6.548 7626355 PMC483918

[88] Villar AV Llano M Cobo M Exposito V Merino R Martin-Duran R. Gender differences of echocardiographic and gene expression patterns in human pressure overload left ventricular hypertrophy. J Mol Cell Cardiol. (2009) 46:526–35. 10.1016/j.yjmcc.2008.12.024 19639678

[89] Villari B Campbell SE Schneider J Vassalli G Chiariello M Hess OM. Sex-dependent differences in left ventricular function and structure in chronic pressure overload. Eur Heart J. (1995) 16:1410–9. 10.1093/oxfordjournals.eurheartj.a060749 8746910

[90] Cleland JG Swedberg K Follath F Komajda M Cohen-Solal A Aguilar JC. The EuroHeart Failure survey programme– a survey on the quality of care among patients with heart failure in Europe. Part 1: patient characteristics and diagnosis. Eur Heart J. (2003) 24:442–63. 10.1016/S0195-668X(02)00823-0 12633546

[91] Garcia R Salido-Medina AB Gil A Merino D Gomez J Villar AV. Sex-specific regulation of miR-29b in the myocardium under pressure overload is associated with differential molecular, structural and functional remodeling patterns in mice and patients with aortic stenosis. Cells. (2020) 9:833. 10.3390/cells9040833 32235655 PMC7226763

[92] Kararigas G Dworatzek E Petrov G Summer H Schulze TM Baczko I. Sex-dependent regulation of fibrosis and inflammation in human left ventricular remodelling under pressure overload. Eur J Heart Fail. (2014) 16:1160–7. 10.1002/ejhf.171 25287281

[93] Gaignebet L Kandula MM Lehmann D Knosalla C Kreil DP Kararigas G. Sex-specific human cardiomyocyte gene regulation in left ventricular pressure overload. Mayo Clin Proc. (2020) 95:688–97. 10.1016/j.mayocp.2019.11.026 31954524

[94] Franconi F Campesi I. Pharmacogenomics, pharmacokinetics and pharmacodynamics: interaction with biological differences between men and women. Br J Pharmacol. (2014) 171:580–94. 10.1111/bph.12362 23981051 PMC3969074

[95] Jochmann N Stangl K Garbe E Baumann G Stangl V. Female-specific aspects in the pharmacotherapy of chronic cardiovascular diseases. Eur Heart J. (2005) 26:1585–95. 10.1093/eurheartj/ehi397 15996977

[96] Rathore SS Wang Y Krumholz HM. Sex-based differences in the effect of digoxin for the treatment of heart failure. N Engl J Med. (2002) 347:1403–11. 10.1056/NEJMoa021266 12409542

[97] Cui C Huang C Liu K Xu G Yang J Zhou Y. Large-scale in silico identification of drugs exerting sex-specific effects in the heart. J Transl Med. (2018) 16:236. 10.1186/s12967-018-1612-6 30157868 PMC6116388

[98] Cramariuc D Rogge BP Lonnebakken MT Boman K Bahlmann E Gohlke-Barwolf C. Sex differences in cardiovascular outcome during progression of aortic valve stenosis. Heart. (2015) 101:209–14. 10.1136/heartjnl-2014-306078 25301859 PMC4316939

[99] Martinez-Selles M Doughty RN Poppe K Whalley GA Earle N Tribouilloy C. Gender and survival in patients with heart failure: interactions with diabetes and aetiology. Results from the MAGGIC individual patient meta-analysis. Eur J Heart Fail. (2012) 14:473–9. 10.1093/eurjhf/hfs026 22402958

[100] Petrov G Dworatzek E Schulze TM Dandel M Kararigas G Mahmoodzadeh S. Maladaptive remodeling is associated with impaired survival in women but not in men after aortic valve replacement. JACC Cardiovasc Imag. (2014) 7:1073–80. 10.1016/j.jcmg.2014.06.017 25306541

[101] Org E Mehrabian M Parks BW Shipkova P Liu X Drake TA. Sex differences and hormonal effects on gut microbiota composition in mice. Gut Microbes. (2016) 7:313–22. 10.1080/19490976.2016.1203502 27355107 PMC4988450

[102] Stanley EG Bailey NJ Bollard ME Haselden JN Waterfield CJ Holmes E. Sexual dimorphism in urinary metabolite profiles of Han Wistar rats revealed by nuclear-magnetic-resonance-based metabonomics. Anal Biochem. (2005) 343:195–202. 10.1016/j.ab.2005.01.024 15993369

[103] Bolnick DI Snowberg LK Hirsch PE Lauber CL Org E Parks B. Individual diet has sex-dependent effects on vertebrate gut microbiota. Nat Commun. (2014) 5:4500. 10.1038/ncomms5500 25072318 PMC4279269

[104] Bridgewater LC Zhang C Wu Y Hu W Zhang Q Wang J. Gender-based differences in host behavior and gut microbiota composition in response to high fat diet and stress in a mouse model. Sci Rep. (2017) 7:10776. 10.1038/s41598-017-11069-4 28883460 PMC5589737

[105] Suzuki Y Ikeda K Sakuma K Kawai S Sawaki K Asahara T. Association between yogurt consumption and intestinal microbiota in healthy young adults differs by host gender. Front Microbiol. (2017) 8:847. 10.3389/fmicb.2017.00847 28553274 PMC5425481

[106] Pei J Harakalova M Treibel TA Lumbers RT Boukens BJ Efimov IR. H3K27ac acetylome signatures reveal the epigenomic reorganization in remodeled non-failing human hearts. Clin Epigenetics. (2020) 12:106. 10.1186/s13148-020-00895-5 32664951 PMC7362435

[107] Iorga A Cunningham CM Moazeni S Ruffenach G Umar S Eghbali M. The protective role of estrogen and estrogen receptors in cardiovascular disease and the controversial use of estrogen therapy. Biol Sex Differ. (2017) 8:33. 10.1186/s13293-017-0152-8 29065927 PMC5655818

[108] Murphy E. Estrogen signaling and cardiovascular disease. Circ Res. (2011) 109:687–96. 10.1161/CIRCRESAHA.110.236687 21885836 PMC3398381

[109] Murphy E Steenbergen C. Estrogen regulation of protein expression and signaling pathways in the heart. Biol Sex Differ. (2014) 5:6. 10.1186/2042-6410-5-6 24612699 PMC3975301

[110] Menazza S Murphy E. The expanding complexity of estrogen receptor signaling in the cardiovascular system. Circ Res. (2016) 118:994–1007. 10.1161/CIRCRESAHA.115.305376 26838792 PMC5012719

[111] Puglisi R Mattia G Care A Marano G Malorni W Matarrese P. Non-genomic effects of estrogen on cell homeostasis and remodeling with special focus on cardiac ischemia/reperfusion injury. Front Endocrinol. (2019) 10:733. 10.3389/fendo.2019.00733 31708877 PMC6823206

[112] Schubert C Raparelli V Westphal C Dworatzek E Petrov G Kararigas G. Reduction of apoptosis and preservation of mitochondrial integrity under ischemia/reperfusion injury is mediated by estrogen receptor beta. Biol Sex Differ. (2016) 7:53. 10.1186/s13293-016-0104-8 27688871 PMC5035458

[113] Mahmoodzadeh S Dworatzek E. The role of 17beta-estradiol and estrogen receptors in regulation of Ca(2+) channels and mitochondrial function in cardiomyocytes. Front Endocrinol. (2019) 10:310. 10.3389/fendo.2019.00310 31156557 PMC6529529

[114] Sickinghe AA Korporaal SJA den Ruijter HM Kessler EL. Estrogen contributions to microvascular dysfunction evolving to heart failure with preserved ejection fraction. Front Endocrinol. (2019) 10:442. 10.3389/fendo.2019.00442 31333587 PMC6616854

[115] Ventura-Clapier R Piquereau J Veksler V Garnier A. Estrogens, estrogen receptors effects on cardiac and skeletal muscle mitochondria. Front Endocrinol. (2019) 10:557. 10.3389/fendo.2019.00557 31474941 PMC6702264

[116] Zhang B Miller VM Miller JD. Influences of sex and estrogen in arterial and valvular calcification. Front Endocrinol. (2019) 10:622. 10.3389/fendo.2019.00622 31620082 PMC6763561

[117] Kararigas G Fliegner D Forler S Klein O Schubert C Gustafsson JA. Comparative proteomic analysis reveals sex and estrogen receptor beta effects in the pressure overloaded heart. J Proteome Res. (2014) 13:5829–36. 10.1021/pr500749j 25406860

[118] Kararigas G Fliegner D Gustafsson JA Regitz-Zagrosek V. Role of the estrogen/estrogen-receptor-beta axis in the genomic response to pressure overload-induced hypertrophy. Physiol Genomics. (2011) 43:438–46. 10.1152/physiolgenomics.00199.2010 21325064

[119] Kararigas G Nguyen BT Jarry H. Estrogen modulates cardiac growth through an estrogen receptor alpha-dependent mechanism in healthy ovariectomized mice. Mol Cell Endocrinol. (2014) 382:909–14. 10.1016/j.mce.2013.11.011 24275180

[120] Kararigas G Nguyen BT Zelarayan LC Hassenpflug M Toischer K Sanchez-Ruderisch H. Genetic background defines the regulation of postnatal cardiac growth by 17beta-estradiol through a beta-catenin mechanism. Endocrinology. (2014) 155:2667–76. 10.1210/en.2013-2180 24731099

[121] Sanchez-Ruderisch H Queiros AM Fliegner D Eschen C Kararigas G Regitz-Zagrosek V. Sex-specific regulation of cardiac microRNAs targeting mitochondrial proteins in pressure overload. Biol Sex Differ. (2019) 10:8. 10.1186/s13293-019-0222-1 30728084 PMC6366038

[122] Duft K Schanz M Pham H Abdelwahab A Schriever C Kararigas G. 17beta-Estradiol-induced interaction of estrogen receptor alpha and human atrial essential myosin light chain modulates cardiac contractile function. Basic Res Cardiol. (2017) 112:1. 10.1007/s00395-016-0590-1 27837311

[123] Lai S Collins BC Colson BA Kararigas G Lowe DA. Estradiol modulates myosin regulatory light chain phosphorylation and contractility in skeletal muscle of female mice. Am J Physiol Endocrinol Metab. (2016) 310:E724–33. 10.1152/ajpendo.00439.2015 26956186 PMC4867308

[124] Mahmoodzadeh S Pham TH Kuehne A Fielitz B Dworatzek E Kararigas G. 17beta-Estradiol-induced interaction of ERalpha with NPPA regulates gene expression in cardiomyocytes. Cardiovasc Res. (2012) 96:411–21. 10.1093/cvr/cvs281 22962310

[125] Nguyen BT Kararigas G Jarry H. Dose-dependent effects of a genistein-enriched diet in the heart of ovariectomized mice. Genes Nutr. (2012) 8:383–90. 10.1007/s12263-012-0323-5 23108595 PMC3689888

[126] Nguyen BT Kararigas G Wuttke W Jarry H. Long-term treatment of ovariectomized mice with estradiol or phytoestrogens as a new model to study the role of estrogenic substances in the heart. Planta Med. (2012) 78:6–11. 10.1055/s-0031-1280228 21928168

[127] Kararigas G Becher E Mahmoodzadeh S Knosalla C Hetzer R Regitz-Zagrosek V. Sex-specific modification of progesterone receptor expression by 17beta-oestradiol in human cardiac tissues. Biol Sex Differ. (2010) 1:2. 10.1186/2042-6410-1-2 21208464 PMC3010101

[128] Kararigas G Bito V Tinel H Becher E Baczko I Knosalla C. Transcriptome characterization of estrogen-treated human myocardium identifies Myosin regulatory light chain interacting protein as a sex-specific element influencing contractile function. J Am Coll Cardiol. (2012) 59:410–7. 10.1016/j.jacc.2011.09.054 22261164

[129] Hein S Hassel D Kararigas G. The zebrafish (Danio rerio) is a relevant model for studying sex-specific effects of 17beta-estradiol in the adult heart. Int J Mol Sci. (2019) 20:6287. 10.3390/ijms20246287 31847081 PMC6940842

[130] Fliegner D Schubert C Penkalla A Witt H Kararigas G Dworatzek E. Female sex and estrogen receptor-beta attenuate cardiac remodeling and apoptosis in pressure overload. Am J Physiol Regul Integr Comp Physiol. (2010) 298:R1597–606. 10.1152/ajpregu.00825.2009 20375266

[131] Queiros AM Eschen C Fliegner D Kararigas G Dworatzek E Westphal C. Sex- and estrogen-dependent regulation of a miRNA network in the healthy and hypertrophied heart. Int J Cardiol. (2013) 169:331–8. 10.1016/j.ijcard.2013.09.002 24157234

[132] Kararigas G. Oestrogenic contribution to sex-biased left ventricular remodelling: the male implication. Int J Cardiol. (2021) 343:83–4. 10.1016/j.ijcard.2021.09.020 34537308

[133] Liu Y Yao Y Li H Qiao F Wu J Du ZY. Influence of endogenous and exogenous estrogenic endocrine on intestinal microbiota in zebrafish. PLoS One. (2016) 11:e0163895. 10.1371/journal.pone.0163895 27701432 PMC5049800

[134] Lindheim L Bashir M Munzker J Trummer C Zachhuber V Leber B. Alterations in gut microbiome composition and barrier function are associated with reproductive and metabolic defects in women with polycystic ovary syndrome (PCOS): a pilot study. PLoS One. (2017) 12:e0168390. 10.1371/journal.pone.0168390 28045919 PMC5207627

[135] Liu R Zhang C Shi Y Zhang F Li L Wang X. Dysbiosis of gut microbiota associated with clinical parameters in polycystic ovary syndrome. Front Microbiol. (2017) 8:324. 10.3389/fmicb.2017.00324 28293234 PMC5328957

[136] Torres PJ Siakowska M Banaszewska B Pawelczyk L Duleba AJ Kelley ST. Gut microbial diversity in women with polycystic ovary syndrome correlates with hyperandrogenism. J Clin Endocrinol Metab. (2018) 103:1502–11. 10.1210/jc.2017-02153 29370410 PMC6276580

[137] Insenser M Murri M Del Campo R Martinez-Garcia MA Fernandez-Duran E Escobar-Morreale HF. Gut microbiota and the polycystic ovary syndrome: influence of sex, sex hormones, and obesity. J Clin Endocrinol Metab. (2018) 103:2552–62. 10.1210/jc.2017-02799 29897462

[138] Harada N Hanaoka R Horiuchi H Kitakaze T Mitani T Inui H. Castration influences intestinal microflora and induces abdominal obesity in high-fat diet-fed mice. Sci Rep. (2016) 6:23001. 10.1038/srep23001 26961573 PMC4785334

[139] Arumugam M Raes J Pelletier E Le Paslier D Yamada T Mende DR. Enterotypes of the human gut microbiome. Nature. (2011) 473:174–80. 10.1038/nature09944 21508958 PMC3728647

[140] Kararigas G Seeland U Barcena de Arellano ML Dworatzek E Regitz-Zagrosek V. Why the study of the effects of biological sex is important. Commentary. Ann Ist Super Sanita. (2016) 52:149–50. 10.4415/ANN_16_02_03 27364386

[141] Chen PB Black AS Sobel AL Zhao Y Mukherjee P Molparia B. Directed remodeling of the mouse gut microbiome inhibits the development of atherosclerosis. Nat Biotechnol. (2020) 38:1288–97. 10.1038/s41587-020-0549-5 32541956 PMC7641989

[142] Tariq R Saha S Furqan F Hassett L Pardi D Khanna S. Prevalence and mortality of COVID-19 patients with gastrointestinal symptoms: a systematic review and meta-analysis. Mayo Clin Proc. (2020) 95:1632–48. 10.1016/j.mayocp.2020.06.003 32753138 PMC7284248

[143] Sultan S Altayar O Siddique SM Davitkov P Feuerstein JD Lim JK. AGA institute rapid review of the gastrointestinal and liver manifestations of COVID-19, meta-analysis of international data, and recommendations for the consultative management of patients with COVID-19. Gastroenterology. (2020) 159:320–34 e27. 10.1053/j.gastro.2020.05.001 32407808 PMC7212965

[144] Hoffmann M Kleine-Weber H Schroeder S Kruger N Herrler T Erichsen S. SARS-CoV-2 cell entry depends on ACE2 and TMPRSS2 and is blocked by a clinically proven protease inhibitor. Cell. (2020) 181:271–80 e8. 10.1016/j.cell.2020.02.052 32142651 PMC7102627

[145] Ritter O Kararigas G. Sex-biased vulnerability of the heart to COVID-19. Mayo Clin Proc. (2020) 95:2332–5. 10.1016/j.mayocp.2020.09.017 33153623 PMC7500880

[146] Kararigas G. Sex-biased mechanisms of cardiovascular complications in COVID-19. Physiol Rev. (2022) 102:333–7. 10.1152/physrev.00029.2021 34698551 PMC8805735

